# Emerging Evidence of Chromosome Folding by Loop Extrusion

**DOI:** 10.1101/sqb.2017.82.034710

**Published:** 2018-05-04

**Authors:** Geoffrey Fudenberg, Nezar Abdennur, Maxim Imakaev, Anton Goloborodko, Leonid A. Mirny

**Affiliations:** 1 Gladstone Institute of Data Science and Technology, University of California, San Francisco, California 94158; 2 Computational and Systems Biology Program, Massachusetts Institute of Technology, Cambridge, Massachusetts 02139; 3 Institute for Medical Engineering and Science (IMES), Massachusetts Institute of Technology, Cambridge, Massachusetts 02139; 4 Department of Physics, Massachusetts Institute of Technology, Cambridge, Massachusetts 02139; 5 These authors contributed equally to this work.

## Abstract

Chromosome organization poses a remarkable physical problem with many biological consequences: How can molecular interactions between proteins at the nanometer scale organize micron-long chromatinized DNA molecules, insulating or facilitating interactions between specific genomic elements? The mechanism of active loop extrusion holds great promise for explaining interphase and mitotic chromosome folding, yet remains difficult to assay directly. We discuss predictions from our polymer models of loop extrusion with barrier elements and review recent experimental studies that provide strong support for loop extrusion, focusing on perturbations to CTCF and cohesin assayed via Hi-C in interphase. Finally, we discuss a likely molecular mechanism of loop extrusion by structural maintenance of chromosomes complexes.

Mammalian interphase chromosomes exhibit both cell type- and locus-specific organizations that manifest characteristic patterns on Hi-C maps. These include square areas of enriched contact frequency along the diagonal, termed topologically associating domains (TADs) (Dixon et al. 2012; Nora et al. 2012), often elaborated with *peaks* at their corners (Rao et al. 2014), *grids* of peaks within and between TADs, and enriched lines or *tracks* of contact frequency emanating from a boundary (Fudenberg et al. 2016) (Fig. 1C; for reviews, see Bonev and Cavalli 2016; Merkenschlager and Nora 2016). We distinguish TADs from compartmental segments of the genome, which also appear as squares along the diagonal of Hi-C maps but differ in that they associate to form a checkered pattern in *cis* and in *trans*. Indeed, TADs, peaks, and tracks have an independent mechanistic origin from the patterns associated with the compartmental segregation of active and inactive chromatin (Schwarzer et al. 2017), and we discuss the interplay of these two mechanisms elsewhere (Nuebler et al. 2017). TAD boundaries are frequently demarcated by binding sites of the transcription factor *CTCF*, and are enriched for the structural maintenance of chromosomes (SMC) complex *cohesin*. Functionally, TADs are believed to demarcate coherent *cis* neighborhoods of gene-regulatory activity and hence are crucial for development (Spielmann and Mundlos 2016). To explain how such neighborhoods can be formed, we put forward a mechanism based on a still-hypothetical process of *loop extrusion*.

Here we present emerging evidence that interphase chromosomes are organized by loop extrusion, an active ATP-dependent process that allows nanometer-size molecular machines to organize chromosomes at much larger scales. We review how loop extrusion by cohesins can explain the formation of TADs, peaks, and tracks visible in interphase Hi-C maps. We then detail specific predictions made by the polymer model of loop extrusion, and discuss recent experimental perturbations to CTCF and cohesin that test these predictions and provide strong support for the loop extrusion mechanism. Although we focus on comparisons to mammalian interphase Hi-C experiments, loop extrusion likely plays important roles in other organisms and parts of the cell cycle. We also discuss imaging experiments, single-molecule experiments, and a possible molecular mechanism of loop extrusion.

## POLYMER MODEL OF LOOP EXTRUSION WITH BARRIER ELEMENTS

We frame our discussion around how we originally implemented the mechanism of loop extrusion limited by directional barriers as a polymer model (Fudenberg et al. 2016). In the process of loop extrusion, loop extruding factors (LEFs) translocate along the chromosomes, holding together progressively more genomically distant loci along a chromosome, thus producing dynamically expanding chromatin loops (see Supplemental Movie 1).

LEF translocation is either halted by encounters with other LEFs or probabilistically halted at specific genomic loci that contain *extrusion barriers*. We assume that if halted only on one side, a LEF may continue to extrude chromatin from its other side. LEFs continue to extrude until they dissociate from the chromatin fiber, releasing the extruded loop, as they dynamically exchange with the nucleoplasm.

**Figure 1.**
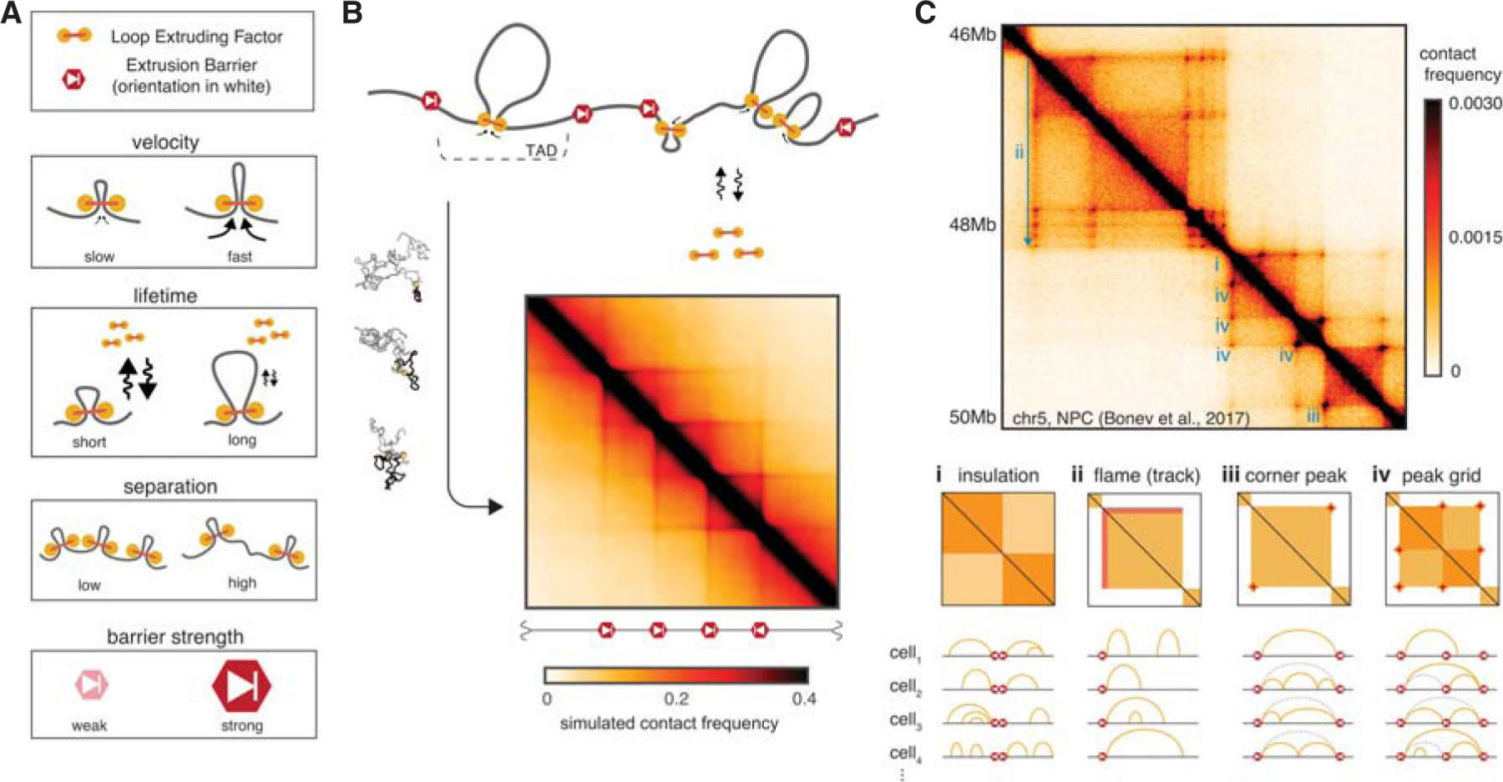
Polymer model of loop extrusion with barrier elements recapitulates features of interphase chromosome folding (see also Supplemental Movie 1). (*A*) Illustrations of the four key parameters governing the dynamics of interphase loop extrusion: LEF velocity, LEF lifetime, LEF separation, and barrier strength. Characterizing how changes to these parameters affect Hi-C maps in silico allows us to make experimental predictions for perturbations. (*B*) To compare our models with Hi-C experiments, we generate ensembles of conformations for each set of parameters, and then compute average contact maps. To compare with imaging experiments, we can calculate other observables (e.g., pairwise distance between loci). (*C*) Interphase Hi-C data from mouse neural progenitor cells (Bonev et al. 2017), plotted with HiGlass (Kerpedjiev et al. 2017), annotated with features that can emerge via loop extrusion in blue (*i–iv*). Arc diagrams depict how stochastic configurations of LEF-mediated loops in distinct nuclei can lead to the population-averaged features. Chromatin loops directly held by LEFs are depicted with yellow arcs, whereas dashed gray arcs depict “transitive loops” from sets of adjacent LEFs. (*i*) Insulation, observed as squares along the diagonal of Hi-C maps (i.e., TADs), arises when extrusion barriers halt LEF translocation. LEFs then facilitate additional contacts within TADs, but not between TADs. (*ii*) Flames (or tracks), observed as straight lines often emerging from the borders of TADs, arise when LEFs become halted on one side at a barrier while continuing to extrude from the other side (referred to as “lines” in Fudenberg et al. 2016). (*iii*) Peaks of enriched contact frequency often appear at the corners of TADs and also often coincide with intersection points of flames. These peaks emerge as a result of LEFs being halted on both sides by extrusion barriers. (*iv*) Peak grids can emerge either when internal boundaries are skipped or via transitive sets of LEF-mediated loops.

The minimal system of LEFs limited by extrusion barriers that we implement is defined by four parameters (Fig. 1A):

- *lifetime* on chromatin (sec)
- *velocity* along the chromatin fiber (kb/sec)
- *separation* between LEFs (kb)
- *permeability* of the extrusion barriers (probability)

For comparison to ensemble-averaged Hi-C experiments, that capture a snapshot of contacts occurring at a particular point in time, it is also useful to define the product of lifetime and velocity, *processivity* (kb), which indicates the average size of a loop that a LEF would extrude if left unobstructed. Motivated by observations of CTCF motif orientations at TAD boundaries and at peaks (Rao et al. 2014; Vietri Rudan et al. 2015), we implement barriers as being *directional*, (i.e., halting LEFs approaching it from only one side). Barriers can be modeled as either halting LEFs as long as the blocking factor is present, or stalling them until LEF dissociation (see Supplemental Movie 1). In our models, the permeability can be thought to represent the probability that a barrier locus is occupied by a blocking factor.

To compare predictions from our simulations with experiments, we generate a simulated ensemble of chromatin conformations for a given set of parameters (Fig. 1B). To accurately capture features of chromatin folding at high resolutions we typically use monomers representing several nucleosomes to simulate 10–50 Mb of chromatin. From these conformations we can extract experimentally relevant observables (Imakaev et al. 2015). These include maps of contact frequency that can be compared to Hi-C contact maps, as well as distributions of spatial distances between pairs of loci, that can be compared with FISH experiments (Fudenberg and Imakaev 2017). From the simulated contact maps, we can then quantify features such as TADs, peaks, and contact frequency decay, as done for experimental Hi-C maps. By comparing simulated and experimental features, we can then define a set of wild-type parameters, from which perturbations, and hence predictions, can be made.

The mechanism of loop extrusion limited by directional barriers recapitulates many features of interphase chromosome folding visible in Hi-C maps (Fig. 1C), including:

- TADs: regions of enriched contact frequency between neighboring barriers
- Tracks: lines emerging from one side of a barrier
- Peaks and grids of peaks, occurring between proximal barriers in *cis* but not between chromosomes
- Presence of inward-oriented CTCF motifs at TAD boundaries and at peak bases

Further support comes from site-specific disruptions of TAD boundaries and peak bases, which respectively result in merging of adjacent TADs (Nora et al. 2012; Narendra et al. 2015; Rodríguez-Carballo et al. 2017) and orientation-dependent losses of peaks (de Wit et al. 2015; Guo et al. 2015; Sanborn et al. 2015). To our knowledge, no alternative mechanism of interphase chromosome organization currently agrees with all the above.

Although we focus here on interphase loop extrusion, we note that loop extrusion by SMCs appears to have important consequences in mitosis (Naumova et al. 2013; Goloborodko et al. 2016a; Gibcus et al. 2018), where the term was coined and first mathematically modeled (Alipour and Marko 2012). The closely related concepts of reeling (Riggs 1990), facilitated tracking (Blackwood and Kadonaga 1998), loop expansion (Kimura et al. 1999) and progressive loop enlargement (Nasmyth 2001) have a rich history. Loop extrusion also appears relevant in bacteria (Gruber 2014; Wang et al. 2015, 2017). There are also related proposals for interphase loop extrusion (Nichols and Corces 2015; Sanborn et al. 2015; Yamamoto and Schiessel 2017; Brackley et al. 2018), which we discuss briefly below.

We note that although the terms “contact,” “loop,” and “interaction” are often used interchangeably in the chromosome organization literature, they are often used to describe very different features of Hi-C contact maps (Forcato et al. 2017). In the context of loop extrusion, we reserve the term “loop” in the very narrow sense of two regions of a continuous chromatin fiber brought together by a LEF at a given point in time. Moreover, simulations (Benedetti et al. 2014; Doyle et al. 2014; Hofmann and Heermann 2015; Fudenberg et al. 2016) and data analyses (Giorgetti et al. 2014; Cattoni et al. 2017; Finn et al. 2017; Fudenberg and Imakaev 2017) show that peaks of contact frequency in interphase Hi-C maps are not consistent with stable chromatin loops. Therefore, we refrain from using “loop” to describe any feature of Hi-C contact maps.

### Challenges for Testing Models of Loop Extrusion

The stochastic nature of loop extrusion poses an experimental challenge for testing predictions from the model. Extruded loops are not directly visible via population-average Hi-C approaches because they are located at different genomic positions in different cells at any given time. Even with single-cell Hi-C methods an individual pair of loci linked by an extruding loop would not appear particularly different from any other captured contact. Visualization of extruded loops by microscopy is similarly challenging due to their continually changing locations both along the genome and in 3D space. Direct confirmation that a particular chromatin loop has been extruded in vivo will require methods that can simultaneously track multiple DNA loci as well as the loop extruders themselves. Nevertheless, much of the strongest evidence to date supporting the role of loop extrusion in interphase comes from changes in Hi-C maps upon perturbations that affect specific components of the loop extrusion machinery.

## PREDICTIONS FROM THE MODEL OF INTERPHASE LOOP EXTRUSION

**Figure 2.**
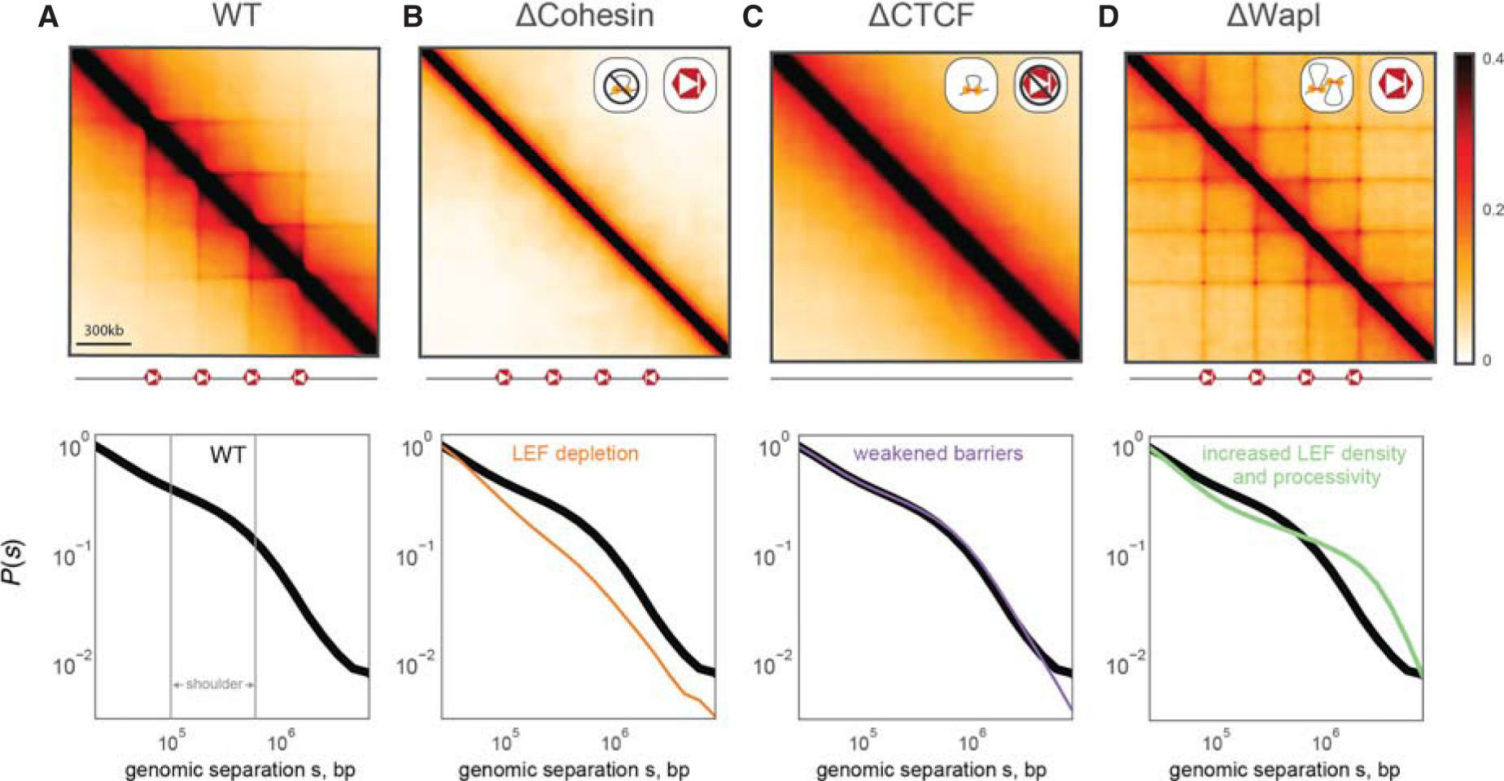
Loop extrusion polymer simulations predict the consequences of cohesin and CTCF perturbations. (*Top* row) Simulated Hi-C maps for indicated perturbations. (*Bottom* row) *P*(*s*) for indicated perturbation compared to WT *P*(*s*). All simulations considered a 36-Mb chain (3600 monomers) with the same positions and orientations of CTCF barriers (separated by 300 kb) and the same LEF velocity (250 3D-per-1D steps). (*A*) WT simulations used processivity 200 kb, separation 200 kb, and barrier strength 0.995. The shoulder in *P*(*s*), indicative of compaction via loop extrusion, is indicated in gray. (*B*) For ΔCohesin, our simulations predict the loss of TADs, peaks, flames, and the shoulder of *P*(*s*). ΔCohesin was simulated using processivity 200 kb, separation 2 Mb, and boundary strength 0.995. This can represent the loss of actively extruding cohesins via ΔNipbl, ΔRad21, or other cohesin subunits. (*C*) For ΔCTCF, our simulations predict the loss of TADs, peaks, flames, yet no discernible change to *P*(*s*). This arises because CTCF plays an instructive role for the activity of extrusion. ΔCTCF was simulated using processivity 200 kb, separation 200 kb, and boundary strength 0.9. (*D*) For ΔWapl, our simulations predict the emergence of additional peaks, including at further genomic separations, as well as an extension of the shoulder in *P*(*s*). ΔWapl was simulated using processivity 1 Mb, separation 150 kb, and boundary strength 0.995.

To make experimental predictions, we must first identify components of the interphase loop extrusion machinery with their biological candidates. Several lines of evidence make us hypothesize that cohesin complexes play the role of LEFs, and CTCF plays the role of an extrusion barrier (Fudenberg et al. 2016). Cohesin is enriched at TAD boundaries in interphase and is highly homologous to condensins, the main complexes responsible for compacting mitotic chromosomes. CTCF is enriched at TAD boundaries at preferentially oriented motifs, and, compared with other transcriptional regulators, binds relatively stably to its cognate sites (for review, see Hansen et al. 2018). With these identities, we discuss how our model of loop extrusion predicts different outcomes for three perturbations: depletion of CTCF, depletion of cohesins, and increased processivity of cohesins (Fig. 2).

### LEF Depletion

For the depletion of the LEF, cohesin, our simulations display two phenomena (Fig. 2B) (i) the loss of TADs and associated Hi-C peaks; and (ii) decompaction of chromatin at the scales of individual extruded loops (<200 kb). Changes in local compaction, in turn, can be studied by observing changes in the contact probability, *P*(*s*), as a function of genomic separation, s. Local compaction is seen as a region of *P*(*s*) with a shallow slope (~100–500 kb), which we refer to as the *shoulder* (Fig. 2A); decompaction leads to reduction or loss of the shoulder region. We note that our models predict that a sharp decrease in LEF processivity would similarly lead to a loss of TADs, peaks, and compaction.

### Extrusion Barrier Depletion

For the depletion of site-specific extrusion barriers, as imposed by CTCF, our simulations also predict the loss of TADs and associated Hi-C peaks (Fig. 2C). However, our simulations predict that other consequences of this perturbation should be very different from depletion of LEFs. This is because in our model, extrusion barriers only impose an instructive function (i.e., their major effect is on the localization of extruded loops rather than on their sizes or abundance). We therefore predict little effect on overall compaction, and hence little change in the *P*(*s*) curve. This differentiates our predictions for CTCF depletion from those for cohesin depletion.

### Increased LEF Density and Processivity

For the depletion of a cohesin unloading factor, like Wapl, our model predicts that the consequent increased processivity and number of LEFs would lead to several phenotypes (Fig. 2D): (i) peaks at corners of TADs become stronger and appear between more distal barrier loci, creating extended grids of peaks; (ii) the orientational preference of barrier loci will become weaker, as LEFs halted at a directional barrier for long durations can stop traffic from the opposing direction as well. Finally, (iii), our model predicts that sufficiently increased coverage by extruded loops will overcompact chromosomes. In Hi-C this would be detected as an extension of the shoulder in *P*(*s*), as opposed to how it recedes in the case of cohesin depletion. Macroscopically, sufficient compaction would cause chromosomes to condense into a prophase-like state with a cohesin-rich central scaffold.

Crucially, our model predicts that the loss of cohesin loop extruders and the loss of CTCF extrusion barriers should both lead to the loss of TADs and Hi-C peaks, yet in completely distinct fashions. Furthermore, increased processivity of cohesin extruders is predicted to manifest distinct phenotypes on Hi-C maps and macroscopic chromosome organization.

## EXPERIMENTAL PERTURBATIONS CONSISTENT WITH INTERPHASE LOOP EXTRUSION

Whereas perturbing CTCF and cohesin dynamics is crucial for testing predictions of loop extrusion, depletion of such essential complexes poses many experimental challenges. For CTCF, cells begin dying after ~4 days of stringent depletion (Nora et al. 2017). For cohesin, there are additional challenges related to its role in sister chromatid cohesion and chromosome segregation during mitosis (Peters and Nishiyama 2012), and its multiple dynamically exchanging subunits and regulators (Peters and Nishiyama 2012; Rhodes et al. 2017) that can be present in different abundances and likely have unique impacts on loop extrusion dynamics. Despite these challenges, recent studies have achieved modulation of cohesin and CTCF that result in dramatic changes, consistent with predictions from polymer models of loop extrusion (Table 1).

### Cohesin Depletion

**Figure 3.**
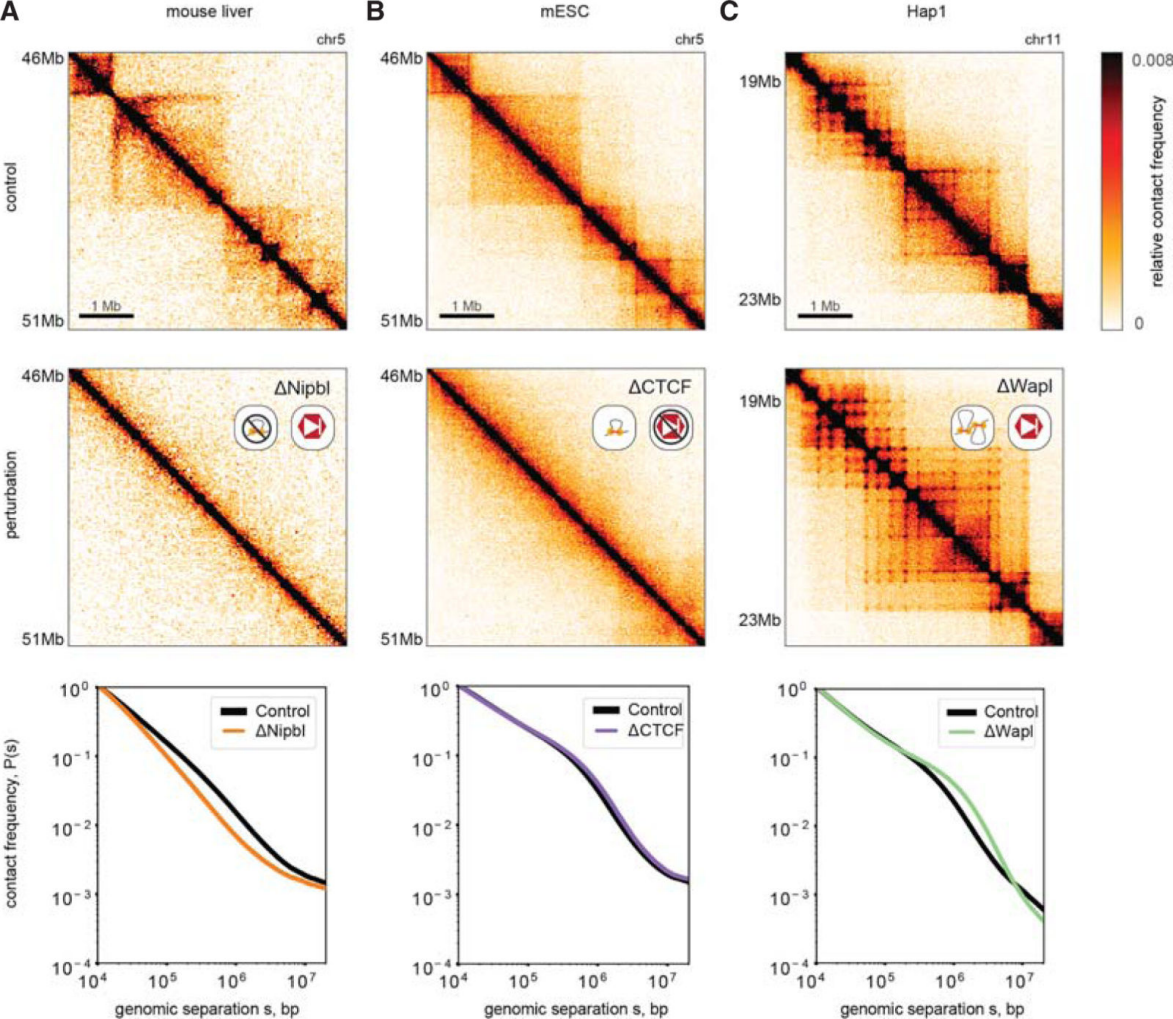
Experimental phenotypes are consistent with predictions from loop extrusion simulations. (*Top* row) Unperturbed experimental Hi-C maps, replotted from indicated studies (see Supplemental Methods; also see interactive HiGlass displays, http://mirnylab.mit.edu/projects/emerging-evidence-for-loop-extrusion). (*Middle* row) Hi-C maps for indicated perturbations. (*Bottom* row) *P*(*s*) for indicated perturbation compared to unperturbed *P*(*s*) normalized to contact frequency at 10 kb. (*A*) Schwarzer et al. (2017) used tissue-specific CRE-inducible gene deletion in mouse liver cells to deplete Nipbl. (*B*) Nora et al. (2017) used an auxin-inducible degron system to deplete CTCF in mESCs. (*C*) Haarhuis et al. (2017) deleted Wapl in the Hap1 haploid human cell line, via CRISPR.

Consistent with our predictions for decreasing the number of active LEFs, depletion of the cohesin loader Nipbl (Scc2) (Schwarzer et al. 2017) and acute degradation of the cohesin kleisin Rad21 (Scc1) (Rao et al. 2017; Wutz et al. 2017) during interphase led to both: (i) complete erasure of TADs and Hi-C peaks (ii) and decompaction, as evidenced by loss of the *P*(*s*) shoulder (Fig. 3A). Decompaction is further supported by imaging, showing loss of H2B clustering by PALM following both RNAi knockdown of NIPBL and AID-mediated degradation of Rad21 (Nozaki et al. 2017). We note that earlier Hi-C studies (Seitan et al. 2013; Sofueva et al. 2013; Zuin et al. 2014) saw limited impact following the depletion of Rad21, potentially due to incomplete depletion.

A corollary of the Nipbl depletion result is that cohesin must be constantly loaded on chromatin to maintain TADs and associated corner peaks. Consistently, TADs and Hi-C peaks are both rapidly lost upon AID-mediated degradation of Rad21 (<3 h (Wutz et al. 2017)) and reestablished after auxin wash-off (40–60 min (Rao et al. 2017)). These consequences follow directly from our loop extrusion models, and the turnover time of cohesin in G1 (~5–30 min [Gerlich et al. 2006; Hansen et al. 2017; Wutz et al. 2017]).

Future studies will be useful to dissect the dynamics of the processes and the potential role of Nipbl beyond that of a loader (Petela et al. 2017; Rhodes et al. 2017). In particular, although Nipbl depletion appears to have a dramatic effect on extrusion, knockout of its cofactor Mau2 (Scc4) appears to have a much weaker effect on loading yet a fairly strong effect on processivity (Haarhuis et al. 2017). Moreover, we note that different components of the interphase extrusion machinery could be limiting at different concentrations and in different contexts. We hypothesize that, via its consequences on loop extrusion, modulation of the levels of various cohesin subunits and interactors can serve to fine-tune overall gene regulation across cell-types and tissues.

### CTCF Depletion

Consistent with our predictions for the loss of site-specific barriers to extrusion, acute auxin-induced degradation of CTCF in mESCs (Nora et al. 2017) and HeLa cells (Wutz et al. 2017) led to a dramatic loss of TADs and Hi-C peaks (Fig. 3B). However, the *P*(*s*) curve did not change, implying that although demarcation of contact-insulating boundaries in Hi-C maps was lost, the same degree of chromatin compaction was maintained. In support of the dynamic exchange of LEFs in our model, the effect of CTCF depletion was fully reversible following a 2-day wash-off period (Nora et al. 2017). We note that stringent dosage depletion was necessary to observe dramatic insulation defects: even a 15% preservation of CTCF showed a relatively mild phenotype (Nora et al. 2017). Similar loss of TADs and peaks were reported in vivo for an inducible CTCF knockout in cardiomyocytes (Lee et al. 2017). Weaker effects have also been reported recently (Kubo et al. 2017; Rosa-Garrido et al. 2017) and earlier (Zuin et al. 2014), but this may have been due to relatively inefficient depletion or lower starting levels of CTCF.

The predicted lack of decondensation following CTCF depletion is further supported by imaging. PALM shows little difference in H2B clustering (Nozaki et al. 2017). Imaging of FISH probes at selected loci upon CTCF degradation show that inter-TAD distances increased, whereas intra-TAD distances remained the same (Nora et al. 2017). Together these results are consistent with global compaction levels being unchanged but with diminished insulation across CTCF sites. Importantly, the lack of chromatin decompaction in CTCF depletion rules out models in which CTCF is strictly required for the loading (Nichols and Corces 2015) of chromatin-bound cohesin and any ensuing cohesin-mediated loops. Instead, the differences in imaging and Hi-C maps upon CTCF versus cohesin depletion are consistent with the loop extrusion model we describe (Fudenberg et al. 2016), in which CTCF barriers serve an instructive function (Wendt and Peters 2009) and cohesin is loaded onto chromatin and can compact chromosomes through extrusion even in the absence of CTCF.

### Wapl Depletion

Consistent with our predictions for increasing the processivity and density of active LEFs, depletion of the cohesin unloader Wapl led to multiple phenotypes observed in Hi-C maps (Gassler et al. 2017; Haarhuis et al. 2017; Wutz et al. 2017) and by imaging (Tedeschi et al. 2013). For Hi-C (Fig. 3C) this includes (i) strengthened peaks at TAD corners, (ii) emergence of new peaks between boundaries at greater separations, creating extended grids of corner peaks; (iii) a weakened correspondence between these features and CTCF motif orientation. Increased local compaction upon Wapl depletion is reflected by (iv) extension of the shoulder in the *P*(*s*) curve and, (v) the emergence of prophase-like vermicelli chromatids via imaging (Tedeschi et al. 2013). This remarkable observation provides further evidence for a *universal molecular mechanism*—loop extrusion—underlying both metaphase and interphase chromosome organization (Imakaev et al. 2015; Dekker and Mirny 2016).

Depletion of another component of the cohesin unloading machinery, Pds5A and Pds5B (Pds5A/B), led to many of the same phenotypes (Wutz et al. 2017). However, there were also intriguing differences that may prove instructive for determining exactly how CTCF halts the progression of cohesin along the chromosome—for example, Pds5 may instruct directional cohesin stalling (Petela et al. 2017; Wutz et al. 2017), and competition between the two HAWK family proteins, Nipbl and Pds5, may regulate cohesin translocation velocity (Petela et al. 2017). The observation that Wapl depletion appears to largely rescue the Hi-C phenotype of Mau2 depletion provides further support to the proposal that the Nipbl/Mau2 “loading complex” also has roles in promoting cohesin processivity for loop extrusion (Haarhuis et al. 2017). Finally, consistent with loop extrusion simulations with increased processivity, the joint depletion of Wapl and Pds5A/B showed even stronger effects in terms of shifting the shoulder in *P*(*s*) and in the emergence of vermicelli.

Collectively, the congruence of both Hi-C and imaging experiments following the perturbation of CTCF and cohesin dynamics strongly supports the role of loop extrusion in interphase. Future simulations and experiments will be valuable for probing the consequences of multiple simultaneous perturbations (Busslinger et al. 2017; Wutz et al. 2017).

## SINGLE-MOLECULE EXPERIMENTS SUPPORTACTIVE LOOP EXTRUSION

Although providing strong support for chromosome folding by loop extension in vivo, the studies discussed above do not directly probe the molecular details of loop extrusion. Molecularly realizing the process of loop extrusion presents a considerable challenge, namely, that the protein complexes performing loop extrusion need to *track consistently* in *cis* along chromatin, over large distances (up to tens-of-thousands of nucleosomes) without falling off. Moreover, the substrate, chromatin, is highly disordered due to nucleosomes and other DNA-bound proteins, likely posing a greater challenge than tracking along microtubules performed by cytoplasmic motors. Here we discuss how recent single molecule experiments argue that loop extrusion likely occurs via an active process, driven by molecular motors. Although many of these observations were made with condensin and bacterial SMCs, they illustrate that loop-extrusion is a plausible mechanism of action for the whole family of SMC proteins, including cohesin.

### ATP-Dependent Translocation

Recently, (Terakawa et al. 2017) showed that a single yeast condensin has motor activity and is able to translocate processively along naked DNA in vitro. Using a DNA curtain assay, they found individual condensin complexes travel unidirectionally, rapidly (~4 kb/min) and processively (~10 kb) in an ATP-dependent manner with 10 nm steps (30 bp on naked DNA). As previous single-molecule studies only reported sliding dynamics of SMCs (Davidson et al. 2016; Kanke et al. 2016; Kim and Loparo 2016; Stigler et al. 2016; for review, see Eeftens and Dekker 2017), the directional translocation observed by Terakawa et al. (2017) is incredibly important.

The high structural homology of cohesin to condensin makes it likely that the same physical mechanism would govern its processive motion, in addition to its established role of mediating sister chromatin cohesion (Peters and Nishiyama 2012). Indeed, the ability of these SMCs to compact chromosomes appears to be remarkably coherent over evolutionary timescales and cellular contexts (Schalbetter et al. 2017). Due to its dual roles, and more elaborate set of subunits, however, reconstituting this activity for cohesin may be more difficult in vitro. Nevertheless, we believe that the in vitro observations of ATP-driven processive condensin translocation argue against the likelihood of motor-free mechanisms (Yamamoto and Schiessel 2017; Brackley et al. 2018) of SMC processivity in general, including for cohesin.

Although strongly supporting the loop extrusion mechanism, the single-molecule experiments leave open several questions of how loop extrusion can work in vivo:

- How can SMCs translocate on chromatinized rather than naked DNA?
- How can translocation result in loop extrusion?
- Is the measured speed of translocation sufficient to generate TADs and peaks?
- Do cells have sufficient ATP budgets to support extrusion during interphase?

### Walking Hypothesis

In particular, it remains to be understood how SMC complexes can translocate on chromatin fibers rather than naked DNA. Translocations while maintaining constant contact with DNA may not always be possible due to the complexity of chromatin fiber and abundance of DNA-bound proteins. Although the size of an SMC complex (~50 nm) exceeds that of a single nucleosome (~10 nm), nucleosomes would constitute challenging obstacles for SMC translocation if maintaining constant contact with DNA is required for translocation.

**Figure 4.**
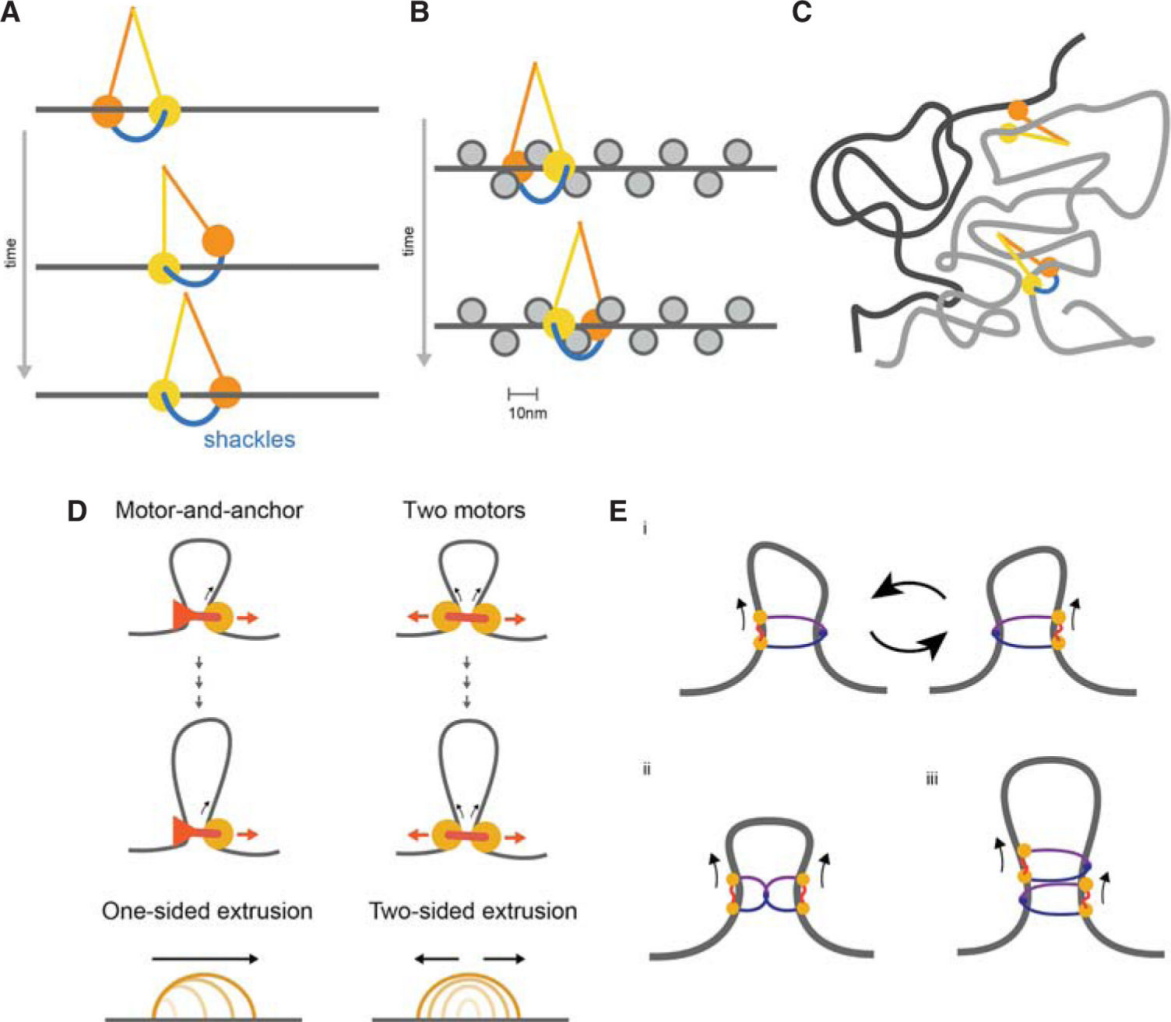
(*A*) Walking as a possible mechanism of SMC translocation, with SMC arms in yellow and orange and kleisin in blue, creating a *shackled walker*. (*B*) Walking along a chromatin fiber, by hopping from linker to linker without disrupting nucleosomal DNA. (*C*) Benefit of topological entrapment: An SMC walker without a kleisin can step from one chromatin strand (gray) to another in its vicinity (black), whereas a shackled SMC walker with a kleisin is able to track in *cis* over long distances. (*D*) Two possible mechanisms for converting translocation to extrusion: The first involves a single translocating motor attached to an anchor, leading to single-sided extrusion; the second involves two motors translocating in opposite directions, leading to two-sided extrusion. (*E*) Possible realizations of motor activity by SMCs (*i–iii*). (*i*) A single SMC acting as single motor that switches between entrapped chromatin strands, effectively performing two-sided extrusion; (*ii*) dimerized SMCs performing two-sided extrusion; (*iii*) alternatively dimerized SMCs performing two-sided extrusion.

A possible solution comes from the structural similarity of SMC domain organization to that of kinesin and myosin motors (Guacci et al. 1993; Peterson 1994) that walk on microtubules and actin, which suggests that SMCs can similarly walk on chromatinized DNA. Importantly, a *walking mechanism* would allow translocation where obstacles such as nucleosomes and other DNA-bound proteins can be passed over, avoiding disruptions of the underlying nucleosomal array. During each step of the walking process, one SMC head can remain DNA-bound, whereas the other hops forward and rebinds nearby DNA (Fig. 4A). SMC walking is consistent with the rapid and flexible dynamics of their arms (Eeftens et al. 2017), and the 10 nm step size (Terakawa et al. 2017) would allow passing over nucleosomes (e.g., by hopping from linker to linker) and other DNA-bound complexes, avoiding the need for unwinding nucleosomal DNA or nucleosome eviction (Fig. 4B).

A walking mechanism would be greatly aided by the known ability of SMCs to *topologically entrap* DNA (Peters and Nishiyama 2012), which can ensure that the walker tracks in *cis*, along the same chromatin fiber (Fig. 4C). Pseudo-topological (Srinivasan et al. 2017) entrapment can similarly help maintain extrusive cohesins on the same DNA molecule over long genomic distances (Kschonsak et al. 2017). In other words, SMC complexes may translocate along the chromatin fiber and accomplish loop extrusion as *shackled walkers* (Fig. 4A).

An important open question is how CTCF, and possibly other chromatin-bound proteins, can halt cohesin translocation whereas nucleosomes do not, when they are fairly similar in size. Although they probed diffusive sliding rather than processive tracking dynamics, Davidson et al. (2016) report that cohesin can rapidly slide over some DNA-bound proteins and nucleosomes, but becomes obstructed by DNA-bound CTCF and transcriptional machinery; a similar, yet more restrictive, dependence of sliding on the size of DNA-bound factors has been reported in other single-molecule studies probing sliding dynamics (Kanke et al. 2016; Stigler et al. 2016). This suggests that CTCF blocks translocation of cohesin by a specific mechanism rather than by steric exclusion—for example, by inhibiting the ATPase action of the cohesin machinery (Petela et al. 2017; Wutz et al. 2017) directly or via other cohesin interactors (e.g., via Pds5) and potentially in concert with cofactors (Hsu et al. 2017). Alternatively, CTCF may recruit additional cofactors to increase its physical size or pose a greater challenge for walking due to its DNA binding geometry (Hashimoto et al. 2017).

### From Translocation to Loop Extrusion

Multiple possibilities exist as to how the translocation of motor complexes along a chromosome can realize the process of loop extrusion. These include (1) a single translocating motor attached to a chromatin anchor; (2) two connected motors translocating in opposite directions; (3) a single motor that switches between two chromatin fiber substrates (Fig. 4D). These architectures for the action of SMC motors can lead to different consequences for the processive dynamics of extrusion. Unidirectional extrusion could result from a single motor-and-anchor architecture. Bidirectional extrusion would emerge from the latter two possibilities. We note there are multiple possibilities for how many SMC complexes are required to realize motor activity (Fig. 4E), either as monomers or, potentially, oligomers (Keenholtz et al. 2017). One advantage of two-motor extrusion is that it naturally allows one motor to continue extruding if the other becomes blocked. Because models discussed here and elsewhere (in Sanborn et al. 2015; Fudenberg et al. 2016; Goloborodko et al. 2016a,b) assumed independent bidirectional extrusion, it remains unclear if one-sided loop extrusion is sufficient to form TADs, peaks, and tracks, as well as to compact mitotic chromatids.

### Velocity of Loop Extruders

The measured rates of stepping and step sizes for condensin (Terakawa et al. 2017) agree well with the expectations of the loop extrusion theory in interphase for cohesin. Using ~2 steps/sec and ~10-nm step size measured in vitro, this gives ~18 kb/min if cohesin moves one nucleosome per step (~150 bp). This is further doubled if cohesin extrusion occurs via a two-motor mechanism, yielding ~36 kb/min. These values are compatible with the ~10–30 kb/min predicted by polymer models as sufficient to generate TADs and corner peaks in vivo. There are several ways to arrive at this estimate. The first involves dividing the size of the largest TADs (~1 Mb [Bonev and Cavalli 2016]) by time to reestablish TADs following exit from mitosis (~0.5–2 h [Naumova et al. 2013], ~30 min [Nagano et al. 2017]) or following auxin wash-off (~30 min [Rao et al. 2017]). Alternatively, one can use the processivity of cohesin estimated from fitting Hi-C data with loop extrusion models (~200–400 kb [Fudenberg et al. 2016]), and divide this by the cohesin turnover time (~5–30 min [Gerlich et al. 2006; Hansen et al. 2017; Wutz et al. 2017]).

We note that pushing by RNA Pol II alone, at its reported velocities, would be too slow (~1.5–3 kb/min [Danko et al. 2013; Jonkers et al. 2014; Veloso et al. 2014]). The observation of cohesin-dependent features in both active and inactive chromatin (Haarhuis et al. 2017; Schwarzer et al. 2017), as well as the transcriptionally inactive maternal zygotic pronucleus (Gassler et al. 2017), further argues against Pol II providing the primary motive force for loop extrusion.

### Energy Budget

A simple estimate shows that the energy burden of ATP consumption by loop-extruding cohesins in interphase is negligible as compared to ATP production in a mammalian cell. Again using 2 ATP per sec per SMC complex (Terakawa et al. 2017), and the total number of actively extruding cohesin molecules, either measured (~100,000 per cell [Hansen AS, pers commun]) or estimated from fitting simulations to Hi-C data (~1 loop-extruder/200 kb, i.e., ~60,000/diploid G2 cell), one obtains a very low rate of ATP consumption (<2 × 10^5^ ATP/sec). This constitutes <0.02% of the 10^9^ ATP/sec production rate by a fibroblast (Flamholz et al. 2014). Thus the energy burden of chromosome organization by cohesin is marginal.

### Direct Observation of Loop Extrusion

While in proofs, a paper (Ganji et al. 2018) appeared that reported a direct observation of loop extrusion in vitro by single purified yeast condensin complexes on DNA. In their experiments, condensins extruded loops of up to tens of kilobases at a speed of up to 1.5 kb/sec in an ATP-dependent fashion. Surprisingly, the extrusion observed was strictly one-sided, which prompts further investigation. Overall, this exciting new study provides the first direct evidence of active loop extrusion by SMC complexes.

## CONCLUSION

Although the key role of molecular motors in the cytoplasm is broadly appreciated (Phillips et al. 2012), there is now a growing appreciation for loop extrusion by SMC complexes as an active processes organizing and compacting chromatin in the nucleus (Haarhuis and Rowland 2017; Nasmyth 2017). Analogous to the myriad uses for the contractile dynamics of active actin and tubulin networks, we hypothesize that interphase loop extrusion has been repurposed for a variety of biological ends (Dekker and Mirny 2016; Fudenberg et al. 2016), including targeting VDJ recombination, and regulation of enhancer–promoter interactions.

Hi-C maps and videos are available at http://mirnylab.mit.edu/projects/emerging-evidence-for-loop-extrusion.

## Supplementary Material

Supplemental Methods

Supplemental Video

Table S1

## Figures and Tables

**Table 1.** List of recent experimental perturbations, prediction from loop extrusion, effects in recent Hi-C experiments, and effect on overall chromatin density

Perturbation^a^	Prediction from loop extrusion	Effect on Hi-C	Effect on compaction
ΔCTCF	Barriers become more permeable	Loss of TADs and peaks, same *P*(*s*) (Nora et al. 2017; Wutz et al. 2017)	Little change overall (Nozaki et al. 2017)
ΔNipbl	Increase separation, possibly decrease velocity	Loss of TADs, peaks and *P*(*s*) shoulder (Schwarzer et al. 2017)	Decompaction (Nozaki et al. 2017)
ΔRad21	Increase separation	Loss of TADs, peaks and *P*(*s*) shoulder (Rao et al. 2017; Wutz et al. 2017; Gassler et al. 2017)	Decompaction (Nozaki et al. 2017)
ΔWapl	Increase processivity, possibly decrease separation	New peaks, extend *P*(*s*) shoulder (Haarhuis et al. 2017; Wutz et al. 2017)	Vermicelli (Tedeschi et al. 2013; Haarhuis et al. 2017; Wutz et al. 2017)

a See Supplemental Table S1 for additional experimental perturbations and details.

## References

[R1] Alipour E, Marko JF. 2012 Self-organization of domain structures by DNA-loop-extruding enzymes. Nucleic Acids Res 40: 11202–11212. 23074191 10.1093/nar/gks925 PMC3526278

[R2] Benedetti F, Dorier J, Burnier Y, Stasiak A. 2014 Models that include supercoiling of topological domains reproduce several known features of interphase chromosomes. Nucleic Acids Res 42: 2848–2855. 24366878 10.1093/nar/gkt1353 PMC3950722

[R3] Blackwood EM, Kadonaga JT. 1998 Going the distance: A current view of enhancer action. Science 281: 60–63. 9679020 10.1126/science.281.5373.60

[R4] Bonev B, Cavalli G. 2016 Organization and function of the 3D genome. Nat Rev Genet 17: 661–678. 27739532 10.1038/nrg.2016.112

[R5] Bonev B, Mendelson Cohen N, Szabo Q, Fritsch L, Papadopoulos GL, Lubling Y, Xu X, Lv X, Hugnot J-P, Tanay A, 2017 Multiscale 3D genome rewiring during mouse neural development. Cell 171: 557–572.e24. 29053968 10.1016/j.cell.2017.09.043 PMC5651218

[R6] Brackley CA, Johnson J, Michieletto D, Morozov AN, Nicodemi M, Cook PR, Marenduzzo D. 2018 Extrusion without a motor: A new take on the loop extrusion model of genome organization. Nucleus 9: 95–103. 29300120 10.1080/19491034.2017.1421825 PMC5973195

[R7] Busslinger GA, Stocsits RR, van der Lelij P, Axelsson E, Tedeschi A, Galjart N, Peters J-M. 2017 Cohesin is positioned in mammalian genomes by transcription, CTCF and Wapl. Nature 544: 503–507. 28424523 10.1038/nature22063 PMC6080695

[R8] Cattoni DI, Cardozo Gizzi AM, Georgieva M, Di Stefano M, Valeri A, Chamousset D, Houbron C, Déjardin S, Fiche J-B, González I, 2017 Single-cell absolute contact probability detection reveals chromosomes are organized by multiple low-frequency yet specific interactions. Nat Commun 8: 1753. 29170434 10.1038/s41467-017-01962-x PMC5700980

[R9] Danko CG, Hah N, Luo X, Martins AL, Core L, Lis JT, Siepel A, Kraus WL. 2013 Signaling pathways differentially affect RNA polymerase II initiation, pausing, and elongation rate in cells. Mol Cell 50: 212–222. 23523369 10.1016/j.molcel.2013.02.015 PMC3640649

[R10] Davidson IF, Goetz D, Zaczek MP, Molodtsov MI, Huis In ‘t Veld PJ, Weissmann F, Litos G, Cisneros DA, Ocampo-Hafalla M, Ladurner R, 2016 Rapid movement and transcriptional re-localization of human cohesin on DNA. EMBO J 35: 2671–2685. 27799150 10.15252/embj.201695402 PMC5167347

[R11] Dekker J, Mirny L. 2016 The 3D genome as moderator of chromosomal communication. Cell 164: 1110–1121. 26967279 10.1016/j.cell.2016.02.007 PMC4788811

[R12] de Wit E, Vos ESM, Holwerda SJB, Valdes-Quezada C, Verstegen MJAM, Teunissen H, Splinter E, Wijchers PJ, Krijger PHL, de Laat W. 2015 CTCF binding polarity determines chromatin looping. Mol Cell 60: 676–684. 26527277 10.1016/j.molcel.2015.09.023

[R13] Dixon JR, Selvaraj S, Yue F, Kim A, Li Y, Shen Y, Hu M, Liu JS, Ren B. 2012 Topological domains in mammalian genomes identified by analysis of chromatin interactions. Nature 485: 376–380. 22495300 10.1038/nature11082 PMC3356448

[R14] Doyle B, Fudenberg G, Imakaev M, Mirny LA. 2014 Chromatin loops as allosteric modulators of enhancer–promoter interactions. PLoS Comput Biol 10: e1003867. 25340767 10.1371/journal.pcbi.1003867 PMC4207457

[R15] Eeftens J, Dekker C. 2017 Catching DNA with hoops-biophysical approaches to clarify the mechanism of SMC proteins. Nat Struct Mol Biol 24: 1012–1020. 29215640 10.1038/nsmb.3507

[R16] Eeftens JM, Bisht S, Kerssemakers J, Kschonsak M, Haering CH, Dekker C. 2017 Real-time detection of condensin-driven DNA compaction reveals a multistep binding mechanism. EMBO J 36: 3448–3457. 29118001 10.15252/embj.201797596 PMC5709735

[R17] Finn E, Pegoraro G, Brandao HB, Valton A-L, Oomen ME, Dekker J, Mirny L, Misteli T. 2017 Heterogeneity and Intrinsic Variation in Spatial Genome Organization. bioRxiv 171801. https://www.biorxiv.org/content/early/2017/08/03/171801. 10.1016/j.cell.2019.01.020 PMC6408223 30799036

[R18] Flamholz A, Phillips R, Milo R. 2014 The quantified cell. Mol Biol Cell 25: 3497–3500. 25368429 10.1091/mbc.E14-09-1347 PMC4230611

[R19] Forcato M, Nicoletti C, Pal K, Livi CM, Ferrari F, Bicciato S. 2017 Comparison of computational methods for Hi-C data analysis. Nat Methods 14: 679–685. 28604721 10.1038/nmeth.4325 PMC5493985

[R20] Fudenberg G, Imakaev M. 2017 FISH-ing for captured contacts: Towards reconciling FISH and 3C. Nat Methods 14: 673–678. 28604723 10.1038/nmeth.4329 PMC5517086

[R21] Fudenberg G, Imakaev M, Lu C, Goloborodko A, Abdennur N, Mirny LA. 2016 Formation of chromosomal domains by loop extrusion. Cell Rep 15: 2038–2049. 27210764 10.1016/j.celrep.2016.04.085 PMC4889513

[R22] Ganji M, Shaltiel IA, Bisht S, Kim E, Kalichava A, Haering CH, Dekker C. 2018 Real-time imaging of DNA loop extrusion by condensin. Science 360: 102–105. 29472443 10.1126/science.aar7831 PMC6329450

[R23] Gassler J, Brandão HB, Imakaev M, Flyamer IM, Ladstätter S, Bickmore WA, Peters J-M, Mirny LA, Tachibana K. 2017 A mechanism of cohesin-dependent loop extrusion organizes zygotic genome architecture. EMBO J 36: 3600–3618. 29217590 10.15252/embj.201798083 PMC5730859

[R24] Gerlich D, Koch B, Dupeux F, Peters J-M, Ellenberg J. 2006 Live-cell imaging reveals a stable cohesin–chromatin interaction after but not before DNA replication. Curr Biol 16: 1571–1578. 16890534 10.1016/j.cub.2006.06.068

[R25] Gibcus JH, Samejima K, Goloborodko A, Samejima I, Naumova N, Nuebler J, Kanemaki MT, Xie L, Paulson JR, Earnshaw WC, 2018 A pathway for mitotic chromosome formation. Science 359: eaao6135. 29348367 10.1126/science.aao6135 PMC5924687

[R26] Giorgetti L, Galupa R, Nora EP, Piolot T, Lam F, Dekker J, Tiana G, Heard E. 2014 Predictive polymer modeling reveals coupled fluctuations in chromosome conformation and transcription. Cell 157: 950–963. 24813616 10.1016/j.cell.2014.03.025 PMC4427251

[R27] Goloborodko A, Imakaev MV, Marko JF, Mirny L. 2016a Compaction and segregation of sister chromatids via active loop extrusion. Elife 5: e14864. 27192037 10.7554/eLife.14864 PMC4914367

[R28] Goloborodko A, Marko JF, Mirny LA. 2016b Chromosome compaction by active loop extrusion. Biophys J 110: 2162–2168. 27224481 10.1016/j.bpj.2016.02.041 PMC4880799

[R29] Gruber S 2014 Multilayer chromosome organization through DNA bending, bridging and extrusion. Curr Opin Microbiol 22: 102–110. 25460803 10.1016/j.mib.2014.09.018

[R30] Guacci V, Yamamoto A, Strunnikov A, Kingsbury J, Hogan E, Meluh P, Koshland D. 1993 Structure and function of chromosomes in mitosis of budding yeast. Cold Spring Harb Symp Quant Biol 58: 677–685. 7956084 10.1101/sqb.1993.058.01.075

[R31] Guo Y, Xu Q, Canzio D, Shou J, Li J, Gorkin DU, Jung I, Wu H, Zhai Y, Tang Y, 2015 CRISPR inversion of CTCF sites alters genome topology and enhancer/promoter function. Cell 162: 900–910. 26276636 10.1016/j.cell.2015.07.038 PMC4642453

[R32] Haarhuis JHI, van der Weide RH, Blomen VA, Yáñez-Cuna JO, Amendola M, van Ruiten MS, Krijger PHL, Teunissen H, Medema RH, van Steensel B, 2017 The cohesin release factor WAPL restricts chromatin loop extension. Cell 169: 693–707.e14. 28475897 10.1016/j.cell.2017.04.013 PMC5422210

[R33] Haarhuis J, Rowland BD. 2017 Cohesin: Building loops, but not compartments. EMBO J 36: 3549–3551. 29217589 10.15252/embj.201798654 PMC5730885

[R34] Hansen AS, Pustova I, Cattoglio Claudia, Tjian Robert, Darzacq X. 2017 CTCF and cohesin regulate chromatin loop stability with distinct dynamics. Elife 6: e25776. 28467304 10.7554/eLife.25776 PMC5446243

[R35] Hansen AS, Cattoglio C, Darzacq X, Tjian R. 2018 Recent evidence that TADs and chromatin loops are dynamic structures. Nucleus 9: 20–32. 29077530 10.1080/19491034.2017.1389365 PMC5990973

[R36] Hashimoto H, Wang D, Horton JR, Zhang X, Corces VG, Cheng X. 2017 Structural basis for the versatile and methylation-dependent binding of CTCF to DNA. Mol Cell 66: 711–720.e3. 28529057 10.1016/j.molcel.2017.05.004 PMC5542067

[R37] Hofmann A, Heermann DW. 2015 The role of loops on the order of eukaryotes and prokaryotes. FEBS Lett 589: 2958–2965. 25912650 10.1016/j.febslet.2015.04.021

[R38] Hsu SC, Gilgenast TG, Bartman CR, Edwards CR, Stonestrom AJ, Huang P, Emerson DJ, Evans P, Werner MT, Keller CA, 2017 The BET protein BRD2 cooperates with CTCF to enforce transcriptional and architectural boundaries. Mol Cell 66: 102–116.e7. 28388437 10.1016/j.molcel.2017.02.027 PMC5393350

[R39] Imakaev MV, Fudenberg G, Mirny LA. 2015 Modeling chromosomes: Beyond pretty pictures. FEBS Lett 589: 3031–3036. 26364723 10.1016/j.febslet.2015.09.004 PMC4722799

[R40] Jonkers I, Kwak H, Lis JT. 2014 Genome-wide dynamics of Pol II elongation and its interplay with promoter proximal pausing, chromatin, and exons. Elife 3: e02407. 24843027 10.7554/eLife.02407 PMC4001325

[R41] Kanke M, Tahara E, Huis In’t P, Nishiyama T. 2016 Cohesin acetylation and Wapl-Pds5 oppositely regulate translocation of cohesin along DNA. EMBO J 35: 2686–2698. 27872142 10.15252/embj.201695756 PMC5167340

[R42] Keenholtz RA, Dhanaraman T, Palou R, Yu J, D’Amours D, Marko JF. 2017 Oligomerization and ATP stimulate condensin-mediated DNA compaction. Sci Rep 7: 14279. 29079757 10.1038/s41598-017-14701-5 PMC5660149

[R43] Kerpedjiev P, Abdennur N, Lekschas F, McCallum C, Dinkla K, Strobelt H, Luber JM, Ouellette SB, Ahzir A, Kumar N, 2017 HiGlass: Web-based visual comparison and exploration of genome interaction maps. bioRxiv 121889. http://biorxiv.org/content/early/2017/03/31/121889. 10.1186/s13059-018-1486-1 PMC6109259 30143029

[R44] Kim H, Loparo JJ. 2016 Multistep assembly of DNA condensation clusters by SMC. Nat Commun 7: 10200. 26725510 10.1038/ncomms10200 PMC4725763

[R45] Kimura K, Rybenkov VV, Crisona NJ, Hirano T, Cozzarelli NR. 1999 13S condensin actively reconfigures DNA by introducing global positive writhe: Implications for chromosome condensation. Cell 98: 239–248. 10428035 10.1016/s0092-8674(00)81018-1

[R46] Kschonsak M, Merkel F, Bisht S, Metz J, Rybin V, Hassler M, Haering CH. 2017 Structural basis for a safety-belt mechanism that anchors condensin to chromosomes. Cell 171: 588–600.e24. 28988770 10.1016/j.cell.2017.09.008 PMC5651216

[R47] Kubo N, Ishii H, Gorkin D, Meitinger F, Xiong X, Fang R, Liu T, Ye Z, Li B, Dixon J, 2017 Preservation of chromatin organization after acute loss of CTCF in mouse embryonic stem cells. bioRxiv 118737. https://www.biorxiv.org/content/early/2017/03/20/118737.

[R48] Lee D, Tan W, Anene G, Li P, Danh T, Tiang Z, Ng SL, Efthymios M, Autio M, Jiang J, 2017 Gene neighbourhood integrity disrupted by CTCF loss in vivo. bioRxiv 187393. https://www.biorxiv.org/content/early/2017/09/12/187393.

[R49] Merkenschlager M, Nora EP. 2016 CTCF and cohesin in genome folding and transcriptional gene regulation. Annu Rev Genomics Hum Genet 17: 17–43. 27089971 10.1146/annurev-genom-083115-022339

[R50] Nagano T, Lubling Y, Várnai C, Dudley C, Leung W, Baran Y, Mendelson Cohen N, Wingett S, Fraser P, Tanay A. 2017 Cellcycle dynamics of chromosomal organization at single-cell resolution. Nature 547: 61–67. 28682332 10.1038/nature23001 PMC5567812

[R51] Narendra V, Rocha PP, An D, Raviram R, Skok JA, Mazzoni EO, Reinberg D. 2015 CTCF establishes discrete functional chromatin domains at the Hox clusters during differentiation. Science 347: 1017–1021. 25722416 10.1126/science.1262088 PMC4428148

[R52] Nasmyth K 2001 Disseminating the genome: Joining, resolving, and separating sister chromatids during mitosis and meiosis. Annu Rev Genet 35: 673. 11700297 10.1146/annurev.genet.35.102401.091334

[R53] Nasmyth K 2017 How are DNAs woven into chromosomes? Science 358: 589–590. 29097534 10.1126/science.aap8729

[R54] Naumova N, Imakaev M, Fudenberg G, Zhan Y, Lajoie BR, Mirny LA, Dekker J. 2013 Organization of the mitotic chromosome. Science 342: 948–953. 24200812 10.1126/science.1236083 PMC4040465

[R55] Nichols MH, Corces VG. 2015 A CTCF code for 3D genome architecture. Cell 162: 703–705. 26276625 10.1016/j.cell.2015.07.053 PMC4745123

[R56] Nora EP, Lajoie BR, Schulz EG, Giorgetti L, Okamoto I, Servant N, Piolot T, van Berkum NL, Meisig J, Sedat J, 2012 Spatial partitioning of the regulatory landscape of the X-inactivation centre. Nature 485: 381–385. 22495304 10.1038/nature11049 PMC3555144

[R57] Nora EP, Goloborodko A, Valton A-L, Gibcus JH, Uebersohn A, Abdennur N, Dekker J, Mirny LA, Bruneau BG. 2017 Targeted degradation of CTCF decouples local insulation of chromosome domains from genomic compartmentalization. Cell 169: 930–944.e22. 28525758 10.1016/j.cell.2017.05.004 PMC5538188

[R58] Nozaki T, Imai R, Tanbo M, Nagashima R, Tamura S, Tani T, Joti Y, Tomita M, Hibino K, Kanemaki MT, 2017 Dynamic organization of chromatin domains revealed by super-resolution live-cell imaging. Mol Cell 67: 282–293.e7. 28712725 10.1016/j.molcel.2017.06.018

[R59] Nuebler J, Fudenberg G, Imakaev M, Abdennur N, Mirny L. 2017 Chromatin organization by an interplay of loop extrusion and compartmental segregation. bioRxiv 2017. https://doi.org/101101/196261. 10.1073/pnas.1717730115 PMC6055145 29967174

[R60] Petela N, Gligoris TG, Metson JS, Lee B-G, Voulgaris M, Hu B, Kikuchi S, Chapard C, Chen W, Rajendra E, 2017 Multiple interactions between Scc1 and Scc2 activate cohesin’s DNA dependent ATPase and replace Pds5 during loading. bioRxiv 205914.

[R61] Peters J-M, Nishiyama T. 2012 Sister chromatid cohesion. Cold Spring Harb Perspect Biol 4: a011130. 23043155 10.1101/cshperspect.a011130 PMC3536341

[R62] Peterson CL. 1994 The SMC family: Novel motor proteins for chromosome condensation? Cell 79: 389–392. 7954805 10.1016/0092-8674(94)90247-x

[R63] Phillips R, Kondev J, Theriot J, Garcia H. 2012 Physical biology of the cell, 2nd edn. Garland Science.

[R64] Rao SSP, Huntley MH, Durand NC, Stamenova EK, Bochkov ID, Robinson JT, Sanborn AL, Machol I, Omer AD, Lander ES, 2014 A 3D map of the human genome at kilobase resolution reveals principles of chromatin looping. Cell 159: 1665–1680. 25497547 10.1016/j.cell.2014.11.021 PMC5635824

[R65] Rao SSP, Huang S-C, Glenn St Hilaire B, Engreitz JM, Perez EM, Kieffer-Kwon K-R, Sanborn AL, Johnstone SE, Bascom GD, Bochkov ID, 2017 Cohesin loss eliminates all loop domains. Cell 171: 305–320.e24. 28985562 10.1016/j.cell.2017.09.026 PMC5846482

[R66] Rhodes J, Mazza D, Nasmyth K, Uphoff S. 2017 Scc2/Nipbl hops between chromosomal cohesin rings after loading. Elife 6: e30000. 28914604 10.7554/eLife.30000 PMC5621834

[R67] Riggs AD. 1990 DNA methylation and late replication probably aid cell memory, and type 1 DNA reeling could aid chromosome folding and enhancer function. Philos Trans R Soc Lond B Biol Sci 326: 285–297. 1968665 10.1098/rstb.1990.0012

[R68] Rodríguez-Carballo E, Lopez-Delisle L, Zhan Y, Fabre PJ, Beccari L, El-Idrissi I, Huynh THN, Ozadam H, Dekker J, Duboule D. 2017 The HoxD cluster is a dynamic and resilient TAD boundary controlling the segregation of antagonistic regulatory landscapes. Genes Dev 31: 2264–2281. 29273679 10.1101/gad.307769.117 PMC5769770

[R69] Rosa-Garrido M, Chapski DJ, Schmitt AD, Kimball TH, Karbassi E, Monte E, Balderas E, Pellegrini M, Shih T-T, Soehalim E, 2017 High-resolution mapping of chromatin conformation in cardiac myocytes reveals structural remodeling of the epigenome in heart failure. Circulation 136: 1613–1625. 28802249 10.1161/CIRCULATIONAHA.117.029430 PMC5648689

[R70] Sanborn AL, Rao SSP, Huang S-C, Durand NC, Huntley MH, Jewett AI, Bochkov ID, Chinnappan D, Cutkosky A, Li J, 2015 Chromatin extrusion explains key features of loop and domain formation in wild-type and engineered genomes. Proc Natl Acad Sci 112: E6456–E6465. 26499245 10.1073/pnas.1518552112 PMC4664323

[R71] Schalbetter SA, Goloborodko A, Fudenberg G, Belton J-M, Miles C, Yu M, Dekker J, Mirny L, Baxter J. 2017 SMC complexes differentially compact mitotic chromosomes according to genomic context. Nat Cell Biol 19: 1071–1080. 28825700 10.1038/ncb3594 PMC5640152

[R72] Schwarzer W, Abdennur N, Goloborodko A, Pekowska A, Fudenberg G, Loe-Mie Y, Fonseca NA, Huber W, Haering CH, Mirny L, 2017 Two independent modes of chromatin organization revealed by cohesin removal. Nature 551: 51–56. 29094699 10.1038/nature24281 PMC5687303

[R73] Seitan VC, Faure AJ, Zhan Y, McCord RP, Lajoie BR, Ing-Simmons E, Lenhard B, Giorgetti L, Heard E, Fisher AG, 2013 Cohesin-based chromatin interactions enable regulated gene expression within preexisting architectural compartments. Genome Res 23: 2066–2077. 24002784 10.1101/gr.161620.113 PMC3847776

[R74] Sofueva S, Yaffe E, Chan W-C, Georgopoulou D, Rudan MV, Mira-Bontenbal H, Pollard SM, Schroth GP, Tanay A, Hadjur S. 2013 Cohesin-mediated interactions organize chromosomal domain architecture. EMBO J 32: 3119–3129. 24185899 10.1038/emboj.2013.237 PMC4489921

[R75] Spielmann M, Mundlos S. 2016 Looking beyond the genes: The role of non-coding variants in human disease. Hum Mol Genet 25: R157–R165. 27354350 10.1093/hmg/ddw205

[R76] Srinivasan M, Scheinost J, Petela N, Gligoris T, Wissler M, Ogushi S, Collier J, Voulgaris M, Kurze A, Chan K-L, 2017 The cohesin ring uses its hinge to organize DNA using non-topological as well as topological mechanisms. bioRxiv 197848. 10.1016/j.cell.2018.04.015 PMC6371919 29754816

[R77] Stigler J, Çamdere GÖ, Koshland DE, Greene EC. 2016 Single-molecule imaging reveals a collapsed conformational state for DNA-bound cohesin. Cell Rep 15: 988–998. 27117417 10.1016/j.celrep.2016.04.003 PMC4856582

[R78] Tedeschi A, Wutz G, Huet S, Jaritz M, Wuensche A, Schirghuber E, Davidson IF, Tang W, Cisneros DA, Bhaskara V, 2013 Wapl is an essential regulator of chromatin structure and chromosome segregation. Nature 501: 564–568. 23975099 10.1038/nature12471 PMC6080692

[R79] Terakawa T, Bisht S, Eeftens JM, Dekker C, Haering CH, Greene EC. 2017 The condensin complex is a mechanochemical motor that translocates along DNA. Science 358: 672–676. 28882993 10.1126/science.aan6516 PMC5862036

[R80] Veloso A, Kirkconnell KS, Magnuson B, Biewen B, Paulsen MT, Wilson TE, Ljungman M. 2014 Rate of elongation by RNA polymerase II is associated with specific gene features and epigenetic modifications. Genome Res 24: 896–905. 24714810 10.1101/gr.171405.113 PMC4032854

[R81] Vietri Rudan M, Barrington C, Henderson S, Ernst C, Odom DT, Tanay A, Hadjur S. 2015 Comparative Hi-C reveals that CTCF underlies evolution of chromosomal domain architecture. Cell Rep 10: 1297–1309. 25732821 10.1016/j.celrep.2015.02.004 PMC4542312

[R82] Wang X, Le TBK, Lajoie BR, Dekker J, Laub MT, Rudner DZ. 2015 Condensin promotes the juxtaposition of DNA flanking its loading site in *Bacillus subtilis*. Genes Dev 29: 1661–1675. 26253537 10.1101/gad.265876.115 PMC4536313

[R83] Wang X, Brandao HB, Le TBK, Laub MT, Rudner DZ. 2017 *Bacillus subtilis* SMC complexes juxtapose chromosome arms as they travel from origin to terminus. Science 355: 524. 28154080 10.1126/science.aai8982 PMC5484144

[R84] Wendt KS, Peters J-M. 2009 How cohesin and CTCF cooperate in regulating gene expression. Chromosome Res 17: 201–214. 19308701 10.1007/s10577-008-9017-7

[R85] Wutz G, Várnai C, Nagasaka K, Cisneros DA, Stocsits RR, Tang W, Schoenfelder S, Jessberger G, Muhar M, Hossain MJ, 2017 Topologically associating domains and chromatin loops depend on cohesin and are regulated by CTCF, WAPL, and PDS5 proteins. EMBO J 36: 3573–3599. 29217591 10.15252/embj.201798004 PMC5730888

[R86] Yamamoto T, Schiessel H. 2017 Osmotic mechanism of the loop extrusion process. Phys Rev E 96: 030402. 29346962 10.1103/PhysRevE.96.030402

[R87] Zuin J, Dixon JR, van der Reijden MIJA, Ye Z, Kolovos P, Brouwer RWW, van de Corput MPC, van de Werken HJG, Knoch TA, van IJcken WFJ, 2014 Cohesin and CTCF differentially affect chromatin architecture and gene expression in human cells. Proc Natl Acad Sci 111: 996–1001. 24335803 10.1073/pnas.1317788111 PMC3903193

