# Supplementary material for: Emerging Evidence of Chromosome Folding by Loop Extrusion
Source: Cold Spring Harb Symp Quant Biol. Author manuscript; Available in PMC 2019 May 13. (PMC6512960; doi:10.1101/sqb.2017.82.034710)
Supplement: Supplemental Methods [file NIHMS1012011-supplement-Supplemental_Methods.docx]

**Methods**

*Hi-C analysis*

Published Hi-C datasets were re-processed using the *Distiller* workflow (<https://github.com/mirnylab/distiller-nf>), producing filtered pairs files (<https://github.com/4dn-dcic/pairix/blob/master/pairs_format_specification.md>) and *Cooler* contact matrix files (<https://github.com/mirnylab/cooler>, [(Abdennur et al. 2017)](https://paperpile.com/c/E9AcEO/eKlZ)). *P(s)* curves were calculated on Hi-C pairs using logarithmically increasing genomic distance bins. For display, filtered bins were imputed via nearest-neighbor interpolation. Interactive *HiGlass* [(Kerpedjiev et al. 2017)](https://paperpile.com/c/E9AcEO/DdmD) displays for relevant datasets are provided at <http://mirnylab.mit.edu/projects/emerging-evidence-for-loop-extrusion>.

*Simulations of loop extrusion with extrusion barriers*

Loop extrusion with barrier element dynamics were modeled as described previously [(Fudenberg et al. 2016)](https://paperpile.com/c/E9AcEO/dqYK), using an updated translocator (<https://bitbucket.org/mirnylab/openmm-polymer/src/8534bc3183e0727a83cdcb9b5525736774035884/examples/loopExtrusion/smcTranslocator.pyx>, described in [(Fudenberg and Imakaev 2017)](https://paperpile.com/c/E9AcEO/Ulv1)). Polymer dynamics were simulated using OpenMM [(Eastman et al. 2013; Eastman and Pande 2010)](https://paperpile.com/c/E9AcEO/Tk3q+AB5u), as described previously [(Fudenberg et al. 2016)](https://paperpile.com/c/E9AcEO/dqYK). All simulations considered a 36Mb chain (3600 monomers) with the same positions and orientations of CTCF barriers (separated by 300kb) and the same LEF velocity (250 3D-per-1D steps). WT simulations used processivity 200kb, separation 200 kb, and pausing barrier strength 0.995. To simulate perturbations, simulations were run with modified parameters. ΔCohesin was simulated using: processivity 200kb, separation 2Mb, and boundary strength 0.995. ΔCTCF was simulated using processivity 200kb, separation 200kb, and boundary strength 0.9. ΔWapl was simulated using processivity 1Mb, separation 150kb, and boundary strength 0.995.

*References**

[Abdennur N, Goloborodko A, Imakaev M, Mirny L. 2017. *mirnylab/cooler v0.7.6*.](http://paperpile.com/b/E9AcEO/eKlZ) <https://zenodo.org/record/1039971>[.](http://paperpile.com/b/E9AcEO/eKlZ)

[Eastman P, Friedrichs MS, Chodera JD, Radmer RJ, Bruns CM, Ku JP, Beauchamp KA, Lane TJ, Wang L-P, Shukla D, et al. 2013. OpenMM 4: A Reusable, Extensible, Hardware Independent Library for High Performance Molecular Simulation. *J Chem Theory Comput* **9**: 461–469.](http://paperpile.com/b/E9AcEO/Tk3q)

[Eastman P, Pande VS. 2010. OpenMM: A Hardware-Independent Framework for Molecular Simulations. *Computing in Science and Engineering* **12**: 34–39.](http://paperpile.com/b/E9AcEO/AB5u)

**References included in this list are cited above but not in the main text.*
