# Supplementary material for: Emerging Evidence of Chromosome Folding by Loop Extrusion
Source: Cold Spring Harb Symp Quant Biol. Author manuscript; Available in PMC 2019 May 13. (PMC6512960; doi:10.1101/sqb.2017.82.034710)
Supplement: Table S1 [file NIHMS1012011-supplement-Table_S1.docx]

| **Perturbation** | **Putative function** | **Study** | **Perturbation method** | **Cell type** | **Reported effects** |
| --- | --- | --- | --- | --- | --- |
| ΔRad21 | extruder subunit | Sofueva et al., 2013 | conditional Cre-mediated deletion (Cre-ERT2 or transfected Cre) | NSCs and ASTs derived from mouse ESC line | Mild effect on Hi-C ma, including relaxed domain structure” |
|  |  | Seitan et al., 2013 | conditional Cre-mediated deletion (CD4-Cre) | Differentiating mouse T-cells | Mild effect on Hi-C map, and resilience of compartment assignments |
|  |  | Zuin et al., 2014 | HRV protease-induced cleavage | HEK239T human hepatocyte cell line | Mild effect on Hi-C map, though with reduced interactions within TADs |
|  |  | Gassler et al., 2017 | conditional Cre-mediated deletion (Zp3-Cre) | Fertilized mouse oocytes | Loss of aggregate TADs and peaks. Loss of P(s) shoulder |
|  |  | Rao et al., 2017 | auxin-inducible degron | HCT-116 human colorectal carcinoma | Loss of TADs and peaks. |
|  |  | Wutz et al., 2017 | auxin-inducible degron | HeLa | Loss of TADs, peaks and P(s) shoulder |
| ΔNipbl | loader | Schwarzer et al., 2017 | conditional tissue-specific Cre-mediated deletion (Ttr-Cre/Esr1) | mouse liver | Loss of TADs, peaks, and P(s) shoulder. |
| ΔMau2 | loading cofactor | Haarhuis et al., 2017 | CRISPR-mediated knockout | HAP1 haploid human cell line | Weakening and shortening of detectable TADs and peaks. P(s) shoulder recedes. |
| ΔCTCF | barrier | Zuin et al., 2014 | RNAi | HEK239T human hepatocyte cell line | Mild effect on Hi-C map, though with an increase in interactions between neighboring domains |
|  |  | Nora et al., 2017 | auxin-inducible degron | E14TG2a mouse ESC line | Loss of TADs and peaks, same *P(s)* |
|  |  | Kubo et al., 2017 (bioRxiv) | auxin-inducible degron | F123 mouse ESC line | Weakened TADs, reduced interaction frequency at peaks |
|  |  | Lee et al., 2017 (bioRxiv) | conditional tissue-specific Cre-mediated deletion (transfected cTnt-Cre) | mouse heart | Loss of TADs & peaks |
|  |  | Rosa-Garrido et al., 2017 | conditional tissue-specific Cre-mediated deletion (MerCreMer) | mouse heart | Loss of some peaks, maintenance of TADs |
|  |  | Wutz et al., 2017 | auxin inducible degron | HeLa | Loss of TADs & peaks, same *P(s)* |
| ΔWapl | unloader | Haarhuis et al., 2017 | CRISPR-mediated knockout | HAP1 haploid human cell line | Increased extent of TADs and peak grids, stronger peaks, *P(s)* shoulder shifts to the right. |
|  |  | Gassler et al., 2017 | conditional Cre-mediated deletion (Zp3-Cre) | Fertilized mouse oocytes | Stronger aggregate TADs and peaks. *P(s)* shoulder shifts to the right |
|  |  | Wutz et al., 2017 | RNAi | HeLa | Increased extent of TADs, peak grids, stronger peaks, *P(s)* shoulder shifts to the right. |
| ΔPds5A/B | unloading cofactor | Wutz et al., 2017 | RNAi | HeLa | Increased extent of TADs, peak grids, stronger peaks, *P(s)* shoulder shifts to the right. |

**Table S1**. List of experimental perturbations, likely consequences for loop extrusion, cell type, and reference where Hi-C for this perturbation was performed.
